# Supplementary material for: Development of a Tetraplex qPCR for the Molecular Identification and Quantification of Human Enteric Viruses, NoV and HAV, in Fish Samples
Source: Microorganisms. 2021 May 27;9(6):1149. doi: 10.3390/microorganisms9061149 (PMC8227966; doi:10.3390/microorganisms9061149)
Supplement: Supplementary file 1 [file microorganisms-09-01149-s001.zip › microorganisms-1219460-supplementary.pdf]

Table S1. Intra-assay variance (repeatability test) using plasmid as standard curve for viral quantification: a) singleplex, b) multiplex.

a)

	NoV GI			NoV GII			HAV			Mengovirus		
Standard (genome copies)[a]	Cq mean (n=3)	SD[b]	CV (%)[c]	Cq mean (*n*=3)	SD[b]	CV (%)[c]	Cq mean (n=3)	SD[b]	CV (%)[c]	Cq mean (n=3)	SD[b]	CV (%)[c]
10^7	13.18	0.05	0.38	12.25	0.03	0.23	11.02	0.11	0.96	23.92	0.29	1.21
10^6	16.84	0.03	0.17	15.89	0.05	0.30	14.60	0.25	1.68	27.55	0.01	0.00
10^5	20.49	0.07	0.36	19.25	0.02	0.08	17.93	0.16	0.89	31.22	0.19	0.62
10^4	24.28	0.04	0.14	22.58	0.13	0.59	21.36	0.17	0.79	34.01	0.33	0.97
10^3	27.85	0.08	0.28	26.01	0.01	0.03	24.79	0.25	0.99			
10^2	31.37	0.34	1.07	29.53	0.19	0.65	28.20	0.23	0.83			
10	34.77	0.02	0.06	32.37	0.30	0.94	31.23	0.56	1.81			

b)

	NoV GI			NoV GII			HAV			Mengovirus		
Standard (genome copies)[a]	Cq mean (n=3)	SD[b]	CV (%)[c]	Cq mean (*n*=3)	SD[b]	CV (%)[c]	Cq mean (n=3)	SD[b]	CV (%)[c]	Cq mean (n=3)	SD[b]	CV (%)[c]
10^7	15.46	0.00	0.00	12.25	0.03	0.23	12.54	0.09	0.70	24.45	0.17	0.68
10^6	18.72	0.00	0.00	15.89	0.05	0.30	15.91	0.06	0.35	28.05	0.11	0.38
10^5	22.48	0.06	0.27	19.25	0.02	0.08	19.26	0.06	0.29	31.37	0.07	0.23
10^4	25.89	0.18	0.68	22.58	0.13	0.59	22.24	0.11	0.51	34.61	0.06	0.18
10^3	29.37	0.06	0.21	26.01	0.00	0.00	25.13	0.00	0.00			
10^2	32.63	0.11	0.32	29.53	0.19	0.65	28.81	0.03	0.09			
10				32.37	0.30	0.94	31.83	0.00	0.00			

a) Mengovirus standard curve ranges from 10^9 to 10^6 genome/rxn.
b) Standard Deviation
c) Coefficient of Variation

Table S2. Inter-assay variance (reproducibility test) using plasmid as standard curve for viral quantification: a) singleplex, b) multiplex.

a)

.

	NoV GI			NoV GII			HAV			Mengovirus		
Standard (genome copies)[a]	Cq mean (n=3)	SD[b]	CV (%)[c]	Cq mean (*n*=3)	SD[b]	CV (%)[c]	Cq mean (n=3)	SD[b]	CV (%)[c]	Cq mean (n=3)	SD[b]	CV (%)[c]
10^7	13.35	0.33	2.49	12.06	0.33	2.73	11.03	0.09	0.80	23.75	0.29	1.22
10^6	17.02	0.25	1.49	15.76	0.15	0.97	14.56	0.18	1.24	27.55	0.01	0.00
10^5	20.50	0.07	0.33	19.50	0.32	1.64	18.00	0.17	0.92	31.33	0.19	0.62
10^4	24.28	0.03	0.12	22.58	0.36	1.58	21.41	0.14	0.66	34.04	0.33	0.97
10^3	27.69	0.27	0.99	25.88	0.22	0.86	24.69	0.24	0.96			
10^2	31.29	0.31	1.00	29.56	0.16	0.53	28.10	0.24	0.87			
10	34.61	0.25	0.73	32.53	0.52	1.60	31.16	0.41	1.32			

b)

	NoV GI			NoV GII			HAV			Mengovirus		
Standard (genome copies)[a]	Cq mean (n=3)	SD[b]	CV (%)[c]	Cq mean (*n*=3)	SD[b]	CV (%)[c]	Cq mean (n=3)	SD[b]	CV (%)[c]	Cq mean (n=3)	SD[b]	CV (%)[c]
10^7	15.75	0.33	2.12	12.29	0.07	0.59	12.72	0.33	2.56	24.35	0.19	0.80
10^6	19.06	0.39	2.05	15.79	0.12	0.74	16.03	0.37	2.34	28.03	0.14	0.49
10^5	22.82	0.39	1.73	19.20	0.07	0.37	19.21	0.12	0.62	31.62	0.32	1.01
10^4	26.29	0.48	1.83	22.54	0.19	0.86	22.39	0.32	1.43	34.67	0.14	0.40
10^3	29.70	0.37	1.25	25.97	0.19	0.72	25.50	0.68	2.67			
10^2	32.93	0.41	1.25	29.45	0.25	0.85	28.97	0.36	1.26			
10				32.46	0.31	0.97	31.63	0.24	0.76			

a) Mengovirus standard curve ranges from 10^9 to 10^6 genome/rxn.
b) Standard Deviation
c) Coefficient of Variation

Table S3. Intra-assay variance (repeatability test) using *in vitro* transcribed RNA as standard curve for viral quantification: a) singleplex, b) multiplex.

a)

	NoV GI			NoV GII			HAV			Mengovirus		
Standard (genome copies)[a]	Cq mean (n=3)	SD[b]	CV (%)[c]	Cq mean (*n*=3)	SD[b]	CV (%)[c]	Cq mean (n=3)	SD[b]	CV (%)[c]	Cq mean (n=3)	SD[b]	CV (%)[c]
10^7	18.71	0.07	0.38	17.12	0.10	0.56	18.87	0.16	0.85	23.92	0.29	1.21
10^6	22.98	0.10	0.43	20.64	0.28	1.34	21.82	0.08	0.36	27.55	0.01	0.00
10^5	26.63	0.08	0.29	24.49	0.06	0.25	24.90	0.03	0.11	31.22	0.19	0.62
10^4	30.24	0.18	0.61	27.32	0.08	0.27	29.42	0.12	0.39	34.01	0.33	0.97
10^3	34.02	0.04	0.12	30.82	0.30	0.96	33.58	0.30	0.88			
10^2				34.62	0.01	0.02	36.53	0.34	0.93			
10				37.48	0.50	1.32						

b)

	NoV GI			NoV GII			HAV			Mengovirus		
Standard (genome copies)[a]	Cq mean (n=3)	SD[b]	CV (%)[c]	Cq mean (*n*=3)	SD[b]	CV (%)[c]	Cq mean (n=3)	SD[b]	CV (%)[c]	Cq mean (n=3)	SD[b]	CV (%)[c]
10^7	21.30	0.12	0.56	18.40	0.06	0.35	19.93	0.02	0.10	25.06	0.21	0.82
10^6	24.83	0.04	0.15	21.95	0.08	0.36	22.80	0.13	0.58	28.81	0.33	1.13
10^5	28.25	0.04	0.15	25.38	0.03	0.10	26.39	0.34	1.30	32.15	0.24	0.73
10^4	31.37	0.01	0.04	28.89	0.07	0.26	29.32	0.12	0.41	35.33	0.35	0.98
10^3	33.74	0.05	0.15	32.17	0.18	0.55	31.82	0.23	0.71			
10^2				34.82	0.20	0.57						
10				36.13	0.01	0.04						

a) Mengovirus standard curve ranges from 10^9 to 10^6 genome/rxn.
b) Standard Deviation
c) Coefficient of Variation

Table S4. Inter-assay variance (reproducibility test) using *in vitro* transcribed RNA as standard curve for viral quantification: a) singleplex, b) multiplex.

a)

	NoV GI			NoV GII			HAV			Mengovirus		
Standard (genome copies)[a]	Cq mean (n=3)	SD[b]	CV (%)[c]	Cq mean (*n*=3)	SD[b]	CV (%)[c]	Cq mean (n=3)	SD[b]	CV (%)[c]	Cq mean (n=3)	SD[b]	CV (%)[c]
10^7	18.71	0.05	0.27	17.13	0.08	0.46	18.83	0.15	0.78	23.75	0.29	1.22
10^6	22.95	0.10	0.44	20.64	0.23	1.10	21.77	0.10	0.44	27.55	0.01	0.00
10^5	26.70	0.14	0.51	24.40	0.19	0.79	24.85	0.09	0.36	31.33	0.19	0.62
10^4	30.04	0.37	1.23	27.56	0.48	1.74	29.50	0.20	0.66	34.04	0.33	0.97
10^3	33.76	0.45	1.34	30.94	0.30	0.95	33.52	0.24	0.71			
10^2				34.38	0.40	1.17	36.49	0.25	0.69			
10				37.60	0.46	1.23						

b)

	NoV GI			NoV GII			HAV			Mengovirus		
Standard (genome copies)[a]	Cq mean (n=3)	SD[b]	CV (%)[c]	Cq mean (*n*=3)	SD[b]	CV (%)[c]	Cq mean (n=3)	SD[b]	CV (%)[c]	Cq mean (n=3)	SD[b]	CV (%)[c]
10^7	21.28	0.11	0.54	18.66	0.26	1.38	19.63	0.24	1.21	25.76	0.48	1.88
10^6	24.87	0.06	0.23	22.08	0.19	0.86	22.63	0.23	1.01	29.04	0.33	1.12
10^5	28.39	0.14	0.48	25.71	0.38	1.49	26.36	0.23	0.86	32.19	0.20	0.63
10^4	31.47	0.28	0.89	29.23	0.47	1.59	29.27	0.37	1.27	35.33	0.41	1.15
10^3	33.86	0.21	0.63	32.41	0.30	0.92	31.81	0.15	0.48			
10^2				35.12	0.39	1.12						
10				36.35	0.39	1.06						

a) Mengovirus standard curve ranges from 10^9 to 10^6 genome/rxn.
b) Standard Deviation
c) Coefficient of Variation
